# Metabolomic Biomarkers for Detection, Prognosis and Identifying Recurrence in Endometrial Cancer

**DOI:** 10.3390/metabo10080314

**Published:** 2020-07-31

**Authors:** Kelechi Njoku, Caroline J.J Sutton, Anthony D. Whetton, Emma J. Crosbie

**Affiliations:** 1 Division of Cancer Sciences, School of Medical Sciences, Faculty of Biology, Medicine and Health, University of Manchester, 5th Floor Research, St Mary’s Hospital, Oxford Road, Manchester M13 9WL, UK; kelechi.njoku@manchester.ac.uk; 2 Stoller Biomarker Discovery Centre, Institute of Cancer Sciences, Faculty of Biology, Medicine and Health, University of Manchester, Manchester M13 9PL, UK; tony.whetton@manchester.ac.uk; 3 School of Biological Sciences, Faculty of Biology, Medicine and Health, University of Manchester, Oxford Road, Manchester M13 9WL, UK; caroline.sutton@student.manchester.ac.uk; 4 Department of Obstetrics and Gynaecology, Manchester University NHS Foundation Trust, Manchester Academic Health Science Centre, Manchester M13 9WL, UK

**Keywords:** endometrial cancer, metabolomics, biomarkers, metabolic profiling

## Abstract

Metabolic reprogramming is increasingly recognised as one of the defining hallmarks of tumorigenesis. There is compelling evidence to suggest that endometrial cancer develops and progresses in the context of profound metabolic dysfunction. Whilst the incidence of endometrial cancer continues to rise in parallel with the global epidemic of obesity, there are, as yet, no validated biomarkers that can aid risk prediction, early detection, prognostic evaluation or surveillance. Advances in high-throughput technologies have, in recent times, shown promise for biomarker discovery based on genomic, transcriptomic, proteomic and metabolomic platforms. Metabolomics, the large-scale study of metabolites, deals with the downstream products of the other omics technologies and thus best reflects the human phenotype. This review aims to provide a summary and critical synthesis of the existing literature with the ultimate goal of identifying the most promising metabolite biomarkers that can augment current endometrial cancer diagnostic, prognostic and recurrence surveillance strategies. Identified metabolites and their biochemical pathways are discussed in the context of what we know about endometrial carcinogenesis and their potential clinical utility is evaluated. Finally, we underscore the challenges inherent in metabolomic biomarker discovery and validation and provide fresh perspectives and directions for future endometrial cancer biomarker research.

## 1. Introduction

Endometrial cancer (EC) is the leading gynaecological malignancy in high-income countries and accounted for over 89,000 deaths worldwide in 2018 [1,2]. Its incidence is rising, year-on-year, in tandem with the escalating prevalence of obesity [3]. In the United Kingdom (UK), over 9000 cases are diagnosed annually with incidence rates increasing by almost three-fifths since the early 1990s [4]. While the demographic shift towards an ageing population and declining rates of hysterectomy for benign gynaecological conditions contribute to these trends, the major cause is the growing prevalence of obesity (body mass index ≥ 30 kg/m^2^) [3,5]. Worldwide, obesity has reached epidemic proportions, having nearly tripled in prevalence between 1975 and 2016 [6,7]. About 40% of the world female population aged 18 years and over are overweight and nearly 50% of endometrial cancers are directly attributable to obesity [6,8].

Post-menopausal bleeding (PMB) is a red-flag symptom of EC and is seen in 90% of women with EC. However, only 5% to 10% of women with PMB have EC [5,9]. Despite advances in cancer diagnostics over the past few decades, little, if any, progress has been made in the diagnostic work-up for EC [5,10]. Symptomatic women in the UK are triaged with a transvaginal ultrasound scan (TVS), a highly sensitive test that is limited by its poor specificity. Depending on the endometrial thickness, around 50% undergo further tests, specifically endometrial biopsy with or without hysteroscopy [5,11]. These procedures are expensive and invasive, and can be painful, especially in nulliparous women, and carry a small risk of life-threatening uterine perforation and other serious complications [5,12]. There is an urgent need for biomarkers based on minimally invasive sampling methodologies that could aid EC diagnosis [13,14]. The ideal test should be simple, robust and accurate. “What simple, non-invasive, painless and convenient tests can be used to detect cancer early?” ranked as the most important research priority for the early detection of cancer in the UK-focused research gap analysis led by the James Lind Alliance partnership, representing patients, carers and health professionals [15].

Over 80% of ECs are low-grade and develop in a hyperplastic endometrium (Bokhmans Type I EC) [16]. The Type I tumours are strongly associated with obesity and have a favourable prognosis, in contrast to the Type II tumours which are high-grade and clinically aggressive [17]. Four novel EC categories have recently been proposed by The Cancer Genome Atlas Research Network (TCGA): polymerase epsilon (*POLE*) ultra-mutated, microsatellite unstable (MSI), copy number low and copy-number high [18], and have been validated in multiple studies [19,20]. It has been postulated that these molecular subtypes could be used to better define prognosis and recurrence risk than traditional risk prediction models based on clinical predictors [20,21]. Treatment of EC is usually surgical (hysterectomy and bilateral salpingo-oophorectomy), although a significant minority are offered conservative management in the form of hormonal manipulations, especially women of childbearing age who wish to preserve their fertility [9]. Decisions about adjuvant chemotherapy and radiotherapy are currently based on traditional pathological parameters that lack precision and therefore deny some women with biologically aggressive disease the opportunity to receive life-saving treatments whilst exposing others to unnecessary harms [5,22]. There is, at present, limited evidence to support the routine use of imaging or biochemical testing in the follow-up for EC due mainly to the lack of reliable monitoring tools [5]. The key questions are therefore how best to identify women at greatest risk of the disease [23] and the development of non-invasive strategies that can aid its detection, prognosis and monitoring for recurrence [10,24].

High-throughput technologies have emerged as important tools for biomarker discovery and validation [25]. The “omic” technologies, in particular, deal with the comprehensive sequencing of DNA (genomics), epigenetic modifications (epigenomics), mRNA (transcriptomics), proteins (proteomics) and metabolites (metabolomics) in biological samples [26,27]. While all these approaches have the potential to advance cancer diagnostics, metabolomics in particular provides an unprecedented opportunity for the identification of clinically relevant biomarkers as it best mirrors the human phenotype [28,29].

EC is attractive for metabolomic profiling for two main reasons. First, there is compelling evidence to suggest that EC develops and progresses in the context of a profound metabolic dysfunction [30,31]. Several metabolic risk factors are linked to EC pathogenesis including obesity, type II diabetes mellitus, polycystic ovary syndrome and metabolic syndrome [23,30]. Of these, obesity is the most consistently cited risk factor and is strongly related to EC, with every 5 unit increase in BMI leading to a 60% increased risk of the disease [32]. The effect of adipose tissue-derived estrogen, unopposed by progesterone, is the most supported underlying pathogenic mechanism for endometrial carcinogenesis, yet insulin resistance undoubtedly plays a central role, acting synergistically with unopposed estrogen to promote tumour growth [33,34]. Second, the juxtaposition of epithelial, stromal and endothelial endometrial cells, endometrial progenitor/stem cells, inflammatory cells including macrophages, natural killer cells and lymphocytes as well as secreted factors and fluids creates a unique intrauterine microenvironment that drives cellular processes [35,36,37,38]. An imbalance in this microenvironment can not only promote EC development and progression, but also influence treatment response and can be captured via metabolic profiling [38].

## 2. Metabolomics and EC Biomarker Discovery

Metabolomics is the large-scale study of the metabolic response of biological systems [39,40]. The human metabolome is a diverse group of low-molecular weight compounds resulting from both endogenous and exogenous processes, the identity of which can be established by the analysis of various biological samples including blood, urine, tissue and saliva [28,41]. The metabolome most closely reflects the human phenotype in health and disease as it is downstream of the genome, transcriptome and proteome, and in effect, summarises these other “omic” technologies [42]. Cancer-related metabolites are by-products of the cellular processes that result from neoplastic transformation and cellular proliferation, as well as from the body’s immunological (inflammatory) response to malignancy [29,43]. Such metabolites differ quantitatively from those resulting from non-malignant cellular events and thus have the potential to serve as biomarkers for cancer detection [44]. The human metabolome is inherently dynamic and it is therefore not surprising that it evolves in tandem with the progressive nature of the malignancy [29]. As such, metabolites have great potential for monitoring treatment response, disease progression and aiding surveillance. Metabolites that can be linked to specific histological or molecular subtypes of EC may be used to direct targeted treatments, while those that discriminate metabolically healthy from unhealthy obesity phenotypes, for example, have promise for EC risk prediction.

**Figure 1:**
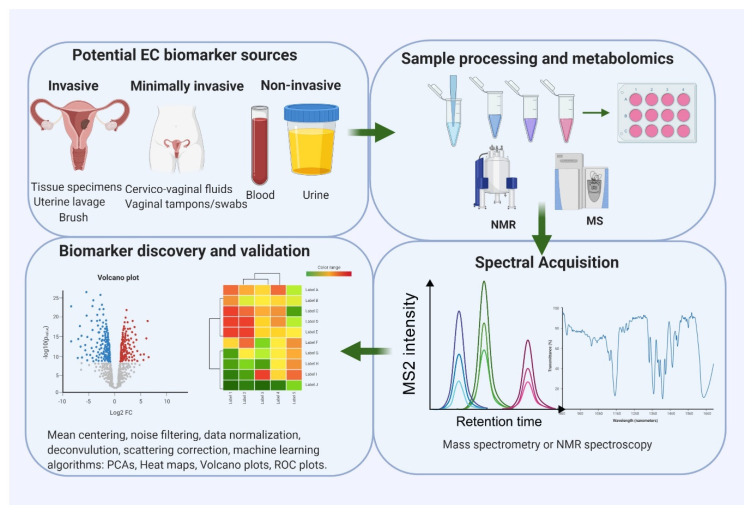
An overview of a typical workflow for endometrial cancer metabolomic biomarker research. Potential sources of endometrial cancer (EC) biomarkers include endometrial tissue specimens, uterine lavage, cervicovaginal specimens, blood and urine. Mass spectrometry (MS) and nuclear magnetic resonance (NMR) analysis are the two main metabolomic platforms for biomarker discovery. Specialised statistical and bioinformatics tools are needed for data analysis, interpretation and integration.

Metabolites for EC detection may be identified in endometrial tissue, brush and lavage specimens, blood samples (serum/plasma) and urine (Figure 1) [45]. Direct sampling of the endometrial cavity by aspiration, brushing and lavage has great potential to yield EC-relevant biomarkers given its close proximity to the tumour. These are, however, invasive to collect, thus limiting their clinical utility [13]. Some metabolites originating in the tumour may find their way into the bloodstream, undergo chemical modification and be excreted in urine [46,47]. Blood-based metabolites are easily accessible but their diagnostic potential is limited by dilution and thus the low yield of cancer-derived metabolites in blood, especially in small and early stage tumours limited to the endometrium [29]. Uterine-shed metabolites may contaminate voided urine samples, particularly in symptomatic women, due to the close proximity of the urethra and vagina, but this may be an unreliable source of biomarkers if uterine shedding is episodic and inconsistent, as might be expected in early stage disease [13]. The anatomical continuity between the upper and lower female genital tracts may provide an opportunity for the collection of uterine tissue-derived EC metabolites using minimally invasive strategies such as vaginal swabs, tampons and cervicovaginal sampling devices [45]. Further studies exploring this possibility are urgently needed.

### Metabolomic Platforms for EC Biomarker Research

Metabolomics may be targeted or untargeted [48]. Targeted approaches are hypothesis-driven and deal with the identification and quantification of a select group of metabolites that are pre-defined [49]. Untargeted approaches comprehensively identify and quantify measurable metabolites in a given sample with no prior hypothesis [40,50]. Targeted approaches have advantages in terms of being more accurate and precise in comparison to untargeted approaches that are prone to high false positive rates resulting from multiple testing of a multitude of variables (type 1 error) [42,43]. Untargeted approaches are therefore often used in the discovery phase of biomarker research, whilst targeted approaches are used for biomarker validation and verification. Advances in high-throughput technologies have led to pseudo-targeted approaches that can combine the strengths of both targeted and untargeted metabolomics [39,40,42].

The platforms commonly used in endometrial cancer metabolomic studies are liquid chromatography-mass spectrometry (LC-MS) and nuclear magnetic resonance spectroscopy (NMR) (Figure 1) [51,52]. LC-MS deals with the ionisation of chromatographically separated analytes and their subsequent identification based on their mass-to-charge ratio and chromatographic retention time [53,54]. Metabolites that ionise efficiently are thus more easily identifiable by MS [55]. In contrast to NMR, LC-MS is very sensitive and can be performed with small clinical samples containing low molar quantities of analytes, thus conferring an advantage in large-scale human studies [54]. The use of capillary electrophoresis coupled to mass spectrometry (CE-MS) is another powerful technique for untargeted metabolomics and has been employed in tumour biomarker discovery. This analytical technique is able to profile highly polar and charged metabolites in biological fluids based on the differential transportation of ions in an electric field and provides complementary metabolic information to LC-MS [56]. NMR, on the other hand, analyses samples by subjecting them to an electromagnetic field and subsequently measuring the characteristic radio waves emitted by each metabolite in response to changes in the magnetic field [54,57]. NMR is highly reproducible and unlike MS, it does not require prior sample separation. Its sensitivity is limited, however, as a smaller range of metabolites can be detected by NMR [54]. Other approaches used in metabolomic research include vibrational spectroscopy which explores vibrations induced to the chemical bonds of metabolites following exposure to electromagnetic radiation [58]. Infrared and Raman spectroscopy are the two main vibrational spectroscopy techniques and respond to different types of vibrations, thus complementing each other [58]. The choice of platform to use in biomarker research is dependent on the focus of the study, the nature of clinical samples available, cost, accessibility and availability of expertise. Effective metabolomic studies often utilise multiple platforms, as no single platform can completely identify and quantify all metabolites in a given sample [51,54,57].

## 3. Challenges and Important Considerations in Metabolomic Biomarker Research

**Figure 2:**
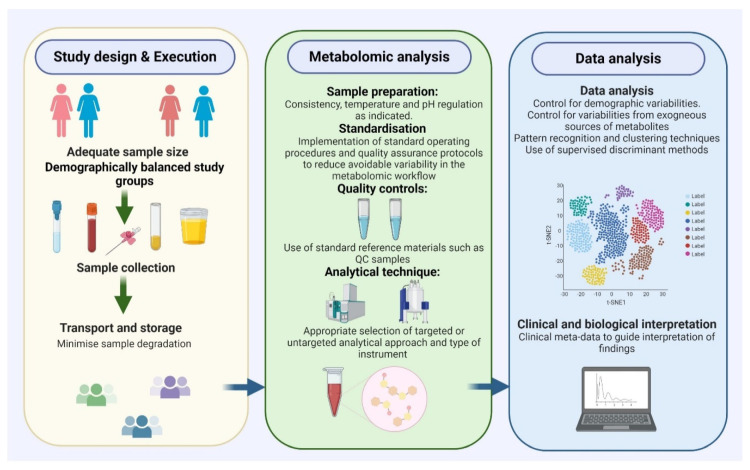
Important considerations for the design of metabolomic biomarker discovery studies. An adequate sample size with demographically balanced study groups, consistency in sample collection, transport, storage and processing as well as use of appropriate analytical techniques are crucial for the identification of viable biomarkers for cancer detection, prognosis and monitoring.

The discovery of viable and clinically relevant metabolite biomarkers for EC is dependent on three main factors: selection of the most appropriate patient groups with adequate sample size, consistent and effective sample preparation and use of appropriate analytical techniques, including statistical methods (Figure 2) [13,27].

### 3.1. Patient Selection

Selecting the most appropriate patients for analysis is crucial for any biomarker discovery or validation study to minimise selection bias [59]. Bias is defined as a systematic deviation from the truth and is an important threat to the validity of biomarker studies [60]. Selection bias occurs when study participants are selected in such a way that samples obtained may not be representative of the population intended to be analysed [61]. Both cases and controls should be as similar as possible except for the condition of interest (EC). Importantly, cases and controls should come from a similar at-risk population. As such, controls for EC diagnostic biomarker studies should include women who are being investigated for PMB but who do not have EC [13]. Where feasible, controls should be matched to cases by demographics such as age, ethnicity, BMI, co-morbidities and lifestyle factors [61]. Participants included as cases should have EC based on gold-standard investigations (histology) to minimise misclassification bias. Another important consideration is sample size, which is closely linked to statistical power and is crucial for the reproducibility of study findings [62]. A power of at least 80% is commonly used as the benchmark for effective biomarker discovery [49,63].

### 3.2. Consistent and Effective Sample Preparation

Ensuring sample integrity is critical for the discovery of viable metabolomic biomarkers [64]. Pre-analytical factors relating to sample collection, storage, transportation and processing can introduce spurious signals into clinical samples thus leading to false positive results [51,64]. As such, samples need to be handled with care and consistency to provide meaningful results. Standard operating procedures with quality control checks should be in place for every step of the analytical process. Exposure of clinical samples to unfavourable conditions that can lead to significant degradation of metabolites should be avoided [28]. As an example, sample preparation techniques will require temperature and pH regulation to ensure that sample metabolites are not altered [64]. Studies based on uterine lavage specimens or tissues may be problematic due to the potential for contamination with blood, especially as PMB is a common symptom of EC. The presence of excessive blood in clinical samples has the potential to distort metabolomic spectral resolution due to the presence of paramagnetic species [65]. It is therefore advisable to wash tissue specimens with saline, although this comes at the risk of potentially removing the tissue metabolic contents [65]. Collection of clinical (fasting status, menopausal status etc.) and other relevant meta-data during sample collection and storage is important to guide the interpretation of metabolomic study results [66]. Time of sample collection needs to be carefully chosen as multiple metabolic processes are under circadian control. Effective sample storage practices, such as the storage of samples in multiple aliquots, should be encouraged as this minimises multiple freeze–thaw cycles which can introduce artefacts into study results [64].

### 3.3. Appropriate Analytical and Statistical Methods

Demographic variability (such as age, BMI, blood pressure, diabetes mellitus) as well as variability from exogenous sources of metabolites (such as food, water, drugs) are important confounders in biomarker research and must be taken into consideration when interpreting results [49]. Use of demographically balanced study groups, exclusion of metabolite markers associated with specific demographics and use of multivariable statistical analyses, including sub-group analyses, are potential methods of dealing with such demographic variabilities [63] Collection of samples after an overnight fast may help to control for exogenous sources of metabolites, such as diet, medications and fluid consumption, and is encouraged [67]. Datasets produced from metabolomic biomarker studies are often highly collinear and noisy, requiring complex analytical methods. Pattern recognition or clustering techniques are needed to reduce dimensionality including (1) unsupervised methods such as principal component analysis (PCA), an adaptive data analysis technique that creates new uncorrelated variables known as principal components that successively maximise variance [68], and (2) supervised methodologies such as orthogonal partial least squares discriminant analysis, which are used to separate these components into predictive and uncorrelated information [63,69]. These techniques provide a visual interpretation of complex datasets and illustrate the degree of separation between study groups.

## 4. Endometrial Cancer Metabolomic Biomarkers in Biofluids

### 4.1. Blood-Based Metabolite Biomarkers

Blood is an ideal source of biomarkers as it is readily available and its use is likely to be acceptable to both clinicians and patients [70]. “Can a blood test be used to detect some or all cancers?” ranked second in our recently completed James Lind Alliance Priority Setting Partnership for Detecting Cancer Early, confirming the appeal of a “cancer blood test” to both clinicians and patients [15]. Several blood-based metabolites have been suggested as potential EC biomarkers and can be broadly classified into amino acids and their derivatives including biogenic amines, acylcarnitines, free fatty acids, phospholipids, sphingolipids, hexoses and hormone metabolites, among others (Table 1) [71,72]. There is, however, insufficient evidence to support the use of any of these metabolites, either singly or in combination, for detection, prognosis or disease monitoring in EC. Evidence for clinical translation is limited by the lack of robust validation of most biomarker candidates and lack of clarity on the mechanistic links underpinning their potential utility as EC biomarkers. Further studies are needed to validate the diagnostic, predictive and prognostic properties of these markers as well as to elucidate the mechanisms underpinning their functions.

#### 4.1.1. Blood-Based Diagnostic Metabolomic EC Biomarkers

A diagnostic biomarker is a biological characteristic that can detect the presence of a disease or condition of interest [73]. The ideal EC diagnostic biomarker should be able to detect ECs of all grades and stages, producing few false positives or negatives [13,74]. An EC metabolic biomarker with adequate diagnostic performance has the potential to not only be used to diagnose symptomatic women but also to screen high-risk asymptomatic women such as those with Lynch syndrome or obesity [75]. Several blood-based EC diagnostic metabolomic biomarkers have been reported in the literature (Table 1) and are mostly by-products of lipids and amino acids. They include acylcholines, monoglycerols, acylcarnitines, phenylalanine, phosphocholine, modified phosphatidylcholine derivatives, lactic acid, progesterone, indole acetic acid, homocysteine, stearic acid, valine, tetradecadienoylcarnitine, 3-hydroxybutyric acid, proline/tyrosine and lyso-platelet-activating factor-16, among others (Table 1). The most commonly reported dysregulated metabolic pathways in the serum of EC cases are lipid- and glycolysis-related pathways [71,76,77]. One of the most promising as a potential biomarker is phosphocholine which has been identified as important in multiple EC studies [77,78] and recently shown to be differentially expressed in the cervicovaginal fluids [79] and tissues [80] of cases versus controls. Audet-Delage and colleagues, using mass spectrometry-based untargeted metabolomics on pre-operative serum samples of 36 EC cases and 18 controls, reported an upregulation of conjugated lipids including acylcholines, monoacylglycerols and acylcarnitines in EC cases and downregulation of free fatty acids, suggesting the potential remodelling of lipid metabolic pathways in EC [71]. Further, they found C5 acylcarnitine 2-methyl butyryl carnitine was elevated in EC cases [71]. These findings align with those of Bahado-Singh, who also reported an upregulation of acylcholines in EC [77]. The mechanism underpinning the role or actions of acylcholines is unclear and further studies are needed. It has been suggested that they may enhance the penetration of oestradiol in tissues, potentiating endometrial carcinogenesis [81]. Acyl-carnitines, on the other hand, are 14-carbon fatty acids attached to a carboxylate through an ester-bond and play crucial roles in mitochondrial fatty acid oxidation [76]. They are reportedly enriched in hypoxic tissues and have previously been linked to breast cancer biochemistry [71,72,82]. Other upregulated lipid metabolites in EC include monoacylglycerols, which are products of the enzymatic hydrolysis of triacylglycerols, and diacylglycerols [71,72,76]. These glycerides are ultimately metabolised (by the action of monoacylglycerol lipase) to free fatty acids, the group of lipid metabolites that are known to be downregulated in EC. The downregulation of the lipase enzyme in EC may, in theory, account for the observed findings [71,83]. Using plasma samples from women with EC (n = 342), atypical hyperplasia (n = 68) and healthy controls (n = 242) in a cross-sectional diagnostic accuracy study, our group was able to show that spectroscopy has the potential to detect EC with 87% sensitivity and 78% specificity [84]. In comparison to the controls, EC cases had an increased lipid-related peak (peak at 1446 cm^−1^) and decreased carbohydrate- and fatty acid-related regions (peaks at 1377 and 900 cm^−1^) [84]. Its diagnostic accuracy was highest for Type I EC and atypical hyperplasia, with sensitivities of 91% and 100%, and specificities of 81% and 88%, respectively [84]. This study was limited by the inability to unpick the exact molecular pathways of identified spectral peaks however, as these peaks can only be tentatively assigned to metabolites because spectral regions may be informed by multiple biological entities [84].

Several amino acids and their derivatives have also been suggested as potential EC diagnostic biomarkers. Amino acids are required for protein synthesis and play crucial roles in maintaining the viability of cancer cells [85,86]. Amino acids also potentiate the redox balance and have been implicated in epigenetic and immune regulatory functions of cancer cells [86]. Ihata and colleagues, in one of the earliest EC metabolic profiling studies evaluated the diagnostic performance of amino acids in the plasma of 80 women with EC, 122 with benign gynaecological diseases and 240 healthy women. They proposed a multiplex model able to distinguish EC cases from control subjects with an AUC of 0.94 [87]. In this study, phenylalanine, valine, histidine, tryptophan, asparagine, serine, leucine and methionine levels were significantly downregulated in EC while isoleucine, ornithine and proline levels were significantly upregulated [87]. All EC cases were matched to healthy controls based on their BMI, suggesting that these changes are unlikely to be related to the effect of overnutrition [87]. The AminoIndex cancer screening test (AICS) is a screening technology that is currently used in Japan for the early detection of multiple malignancies including endometrial/ovarian cancers [88,89,90]. This test uses plasma-free amino acid concentrations to estimate the risks for several malignancies simultaneously with subjects ranked as A, B or C in order of increasing risk of each cancer [89]. In a clinical validation study of AICS, Mikami and colleagues reported a 50% rank C sensitivity for the development of uterine/ovarian cancer within a year and a 33.3% sensitivity within the maximum follow-up period of 6.2 years [89]. It has been reported that AICS test values significantly decrease following resection of malignancies, suggesting that they may not only be viable tools for cancer early detection but also for monitoring treatment response [91]. Shi and colleagues, in a serum metabolomic study of 46 early-stage EC cases and 46 healthy controls, reported phenylalanine, indole acrylic acid, phosphocholine and lyso-platelet-activating factor-16 (lyso-PAF) as discriminatory biomarkers in EC [92]. Further mechanistic studies demonstrated Indoleacrylic acid, lyso-PAF and phenylalanine to exhibit dose-dependent inhibition of tumour cell invasion and migration and suppression of cell proliferation [92]. Of the top ten diagnostic biomarkers identified by Audet-Delage and colleagues, five were peptides and amino acids. Of these, spermine and isovalerate were the most relevant [71]. There was a positive 7-fold change in spermine levels (*p* = 0.0004) in EC cases and a negative 2-fold change in the levels of isovalerate (*p* = 0.015) [71]. The combination of spermine and isovalerate resulted in an overall AUC of 0.875 (95%CI 0.78–0.96) and an age-adjusted AUC of 0.914 (85% CI 0.83–0.99) [71]. A more comprehensive model including spermine, isovalerate, glycylvaline and gamma-glutamyl-s-aminobutyrate resulted in an age-adjusted AUC of 0.92 (95% CI 0.83–1.00) [71]. Spermine is a polyamine closely associated with nucleic acids and thought to stabilise their polymeric helical structure while isovalerate is a product of leucine, isoleucine and valine metabolism [76]. Further studies are needed to elucidate their roles in EC tumorigenesis. Troisi and colleagues, using gas chromatography-mass spectrometry on serum samples of 88 women with EC and 80 healthy controls (discovery phase) and 30 EC and 90 controls (verification phase) investigated the diagnostic performance of serum metabolomic signatures and suggested two models with accuracy that ranged from 62% to 99% for Model I and from 67% to 100% for Model II [93]. An ensemble model based on both models showed an accuracy of 100%. In this study, the most relevant metabolites in class discrimination were lactic acid, progesterone, homocysteine, 3-hydroxybutyrate, linoleic acid, stearic acid, myristic acid, threonine and valine [93]. The accumulation of lactic acid from dysregulated glycolysis (Warburg effect) due to cancer metabolism has been recognised as far back as 1920 [94,95]. Lactic acid has also been shown to promote angiogenesis and to modulate the tumour microenvironment [96]. The finding of lower concentrations of progesterone in EC cases is in keeping with its anti-estrogenic effect [76]. Homocysteine, a homologue of the amino acid cysteine, is involved in maintaining the stability of the DNA and elevated levels have been linked to epithelial malignancies [97], while hydroxybutyrate, a product of acetyl-CoA and an important source of energy during the starvation phase when blood glucose levels are low, has also been linked to EC diagnosis and stage [76]. Further studies are needed to validate these markers before translation into clinical use.

Knific and colleagues, using electrospray ionisation–tandem mass spectrometry on plasma samples obtained from 61 women with EC and 65 controls quantified 163 metabolites [98]. They reported phosphatidylcholines to be significantly downregulated in EC. A diagnostic model incorporating the ratio between acylcarnitine C16 and phosphatidylcholine C40:1, the ratio between proline and tyrosine and the ratio between the two phosphatidylcholines C42:0 and C44:5 demonstrated a sensitivity of 85.3%, specificity of 69.2% and AUC of 0.84 [98]. The accuracy of the model was further improved following the addition of smoking status, an EC protective risk factor (AUC = 0.855) [98]. Further studies are needed to validate these study findings.

Multiple studies have explored the potential of estrogens, their precursors and metabolites to serve as EC diagnostic biomarkers [99,100,101,102,103]. Brinton and colleagues, in a case–control study nested within the Women’s Health Initiative Observational Study, compared baseline serum samples from 313 EC cases and 354 matched controls, all of whom were non-current hormone users and reported estrone and estradiol to be strongly related to EC risk [99]. The association was strongest for unconjugated estradiol (odds ratio fifth vs. first quintile = 6.19 (2.95–13.03), *P*_trend_ = 0.0001) [99]. These findings align with those of Audet-Walsh, who reported an up to 3-fold elevation in estrogen levels in women with Type 1 EC [100]. When used alongside other metabolites in a multimarker panel, circulating endogenous estrogens and their metabolites have enormous potential as EC diagnostic markers and further studies are needed. Blood-based metabolites have been compared to EC risk factors for the prediction of EC. Bahado-Singh and colleagues reported changes in the serum metabolome of EC patients to be superior to BMI for EC prediction and that the addition of BMI to the diagnostic metabolomic panels did not significantly improve the accuracy for EC detection [77].

#### 4.1.2. Blood-Based Predictive and Prognostic Metabolomic EC Biomarkers

Predictive biomarkers are biomarkers that indicate likely response to a particular treatment and can aid decision-making in clinical practice. Prognostic markers, on the other hand, are used to indicate a patient’s overall outcome, independent of therapy [60]. Prognostic factors currently used in clinical practice include histological subtype, tumour grade and stage, depth of myometrial invasion and presence or absence of lymphovascular space invasion (LVSI) [10,104]. Women with high-grade, advanced-stage, non-endometrioid EC generally have poor outcomes [21]. Currently, characterisation of tumour grade and histological type is performed by histopathologists, a subjective process with only moderate inter-observer reproducibility. Markers to improve the prognostic characterisation of EC are thus needed. Recent molecular classification of EC by the TCGA and its validation by other groups has refined the prognostic categorisation of EC [20]. However, it is expensive to complete, requiring multiple workstreams and technologies. Metabolomic markers linked to the four TCGA prognostic categories may be a cheaper alternative for prognostic stratification in EC [76].

Several metabolites have potential for establishing EC histological subtype. They include bradykinin, heme, lactic acid, homocysteine, linoleic acid, myristic acid, progesterone, valine, threonine, stearic acid, 3-hydroxybutyrate, choline, sarcosine, glycine and sulphated androgenic steroids. In the study by Audet-Delage and colleagues, 98 metabolites were differentially expressed between Type I and Type II EC, with 30 metabolites having a higher expression in Type I and 68 having a lower expression [71]. The most promising biomarkers were bradykinin, which showed a 2-fold increase in Type I EC (fold-change = 2.70, *p* = 0.003), and heme, which showed a 4.5-fold increase in Type II EC [71]. Bradykinin is a 9-amino acid residue peptide that acts as a vasodilator and promotes inflammation. It activates phospholipase D in EC [76,105]. Its upregulation is therefore consistent with a pro-inflammatory state, one of the putative biological mechanisms underpinning EC carcinogenesis. Heme, on the other hand, an iron-containing porphyrin, is involved in oxygen transport and energy production and is known to modulate the tumour microenvironment [106]. Other promising prognostic metabolomic biomarkers include saturated long-chain acylcarnitines, which have been reported to be higher in Type II EC, with C20, C24 and C26 acylcarnitines exhibiting a 1.32-, 1.33- and 1.38-fold change, respectively [71]. Acetycarnitine plays important roles in the transport of fatty acids through the mitochondrial membrane during beta-oxidation. Elevated levels are related to dysregulated beta-oxidation with associated increased energy consumption and lipolysis [76]. Further studies are needed to validate these findings. In an infra-red spectroscopy study by Pareskavaidi and colleagues, amide spectral peaks at 1693 cm^−1^ and 1547 cm^−1^ were statistically significantly upregulated in Type II EC compared to Type I, highlighting the potential of blood-based spectroscopy to aid EC stratification [84].

Some metabolites have been associated with the depth of myometrial invasion, specifically hydroxysphingomyelins, phosphatidylcholines and endogenous estrogen metabolites [98]. In the study by Knific and colleagues, a prognostic model for deep myometrial invasion was developed based on the ratio between two hydroxysphingomyelins, SMOH C14:1 and SMOH C24:1, and the ratio between two phosphatidylcholines, PC C40:2 and PC C42:6 [98]. This model demonstrated a sensitivity of 81.3%, specificity of 86.4% and AUC of 0.86 [98]. Sphingolipids are bio-effector molecules implicated in cell growth, proliferation and anti-cancer therapeutics [107], whilst phospholipids form the bilayer components of cellular membranes and are involved in malignant transformation and tumour progression [108]. Metabolomic biomarkers associated with LVSI include hexadecadienyl carnitine, phosphatidylcholine with diacyl residue sum C38:1, phosphatidylcholine with diacyl residue sum C34:4 and phosphatidylcholine with acyl-alkyl residue sum C38:3 [76]. A prognostic model incorporating the ratio between two phosphatidylcholines, PC C34:4 and PC C38:3, and the ratio between acylcarnitine C16:2 and phosphatidylcholine PC C38:1 demonstrated a sensitivity of 88.9%, specificity of 84.3% and AUC of 0.94 for LVSI [98]. Audet-Walsh and colleagues explored the potential of endogenous estrogens and their metabolites to serve as EC prognostic biomarkers and reported 2-Methoxyestradiol-glucoronide (2-MeOE2-3G) to be upregulated in EC cases with deep myometrial invasion, while Estrone sulphate was downregulated in cases with LVSI [100]. The 2-Methoxy metabolite of estradiol has been shown to exhibit anti-angiogenic and pro-apoptotic properties [109,110]. It is postulated that the glucuronidation process that inactivates 2-methoxy estradiol is potentiated in invasive tumours, thus allowing for tumour progression [109].

Several metabolomic biomarkers are associated with EC recurrence, including spermine, acylcholines, sphingolipids, linoleic acid, myristic acid, intermediates from the branched-chain amino acid pathway, polyamines, acylcarnitines, monoacylglycerols, bradykinin, sulfated androgens, heme, bile acids and ceramides [71,76]. Bile acids, for example, are pro-inflammatory and potentiate myometrial sensitivity to hormones as well as modulating cholesterol homeostasis, a process known to drive EC progression [71]. Other metabolites linked to recurrence include estrone sulphate [100] and the panel of 2-oleoyl-glycerol and triacylglycerol-fatty acids (TAG42:2-FA12:0), which demonstrated an AUC = 0.877 (95% CI = 0.730–0.990) for the discrimination of recurrent EC from non-recurrent EC and an age-adjusted AUC_adj_ = 0.901 (95% CI = 0.796–1.000) [71].

Strand and colleagues reported methionine sulfoxide to be strongly associated with poor survival in a study of 40 cases of EC [111]. They proposed a prognostic signature of metabolites found to have an AUC of 0.82–0.98 for EC survival (*p* < 0.001). Methionine sulfoxide is an oxidised form of methionine, an essential amino acid and known precursor of succinyl-CoA, homocysteine and carnitine, among others. Elevated methionine sulfoxide is linked to biological ageing. A dysfunction in its reductase enzyme is linked to cell proliferation, degradation of extracellular matrix and cancer progression [112]. This marker is yet to be externally validated and further studies are needed.

**Table 1:** Most promising blood-based EC metabolomic biomarker candidates and their functions.

Metabolite	Group/Sub-Class	Potential Clinical Utility	Biochemical Function and Summary of Evidence
2-oleoylglycerol [71]	Conjugated lipids	Prognosis Monitor disease recurrence	Produced by lipolysis. Have signalling functions. Activate G-protein-coupled GPR119.
3-hydroxybutyrate [77,93]	Fatty acid metabolite	Diagnosis/early detection	Marker of mitochondrial fatty acid beta-oxidation. Synthesised in the liver from acetyl-CoA. Source of energy during low glucose levels.
Acylcarnitines [71,72]	Conjugated lipids (Fatty acyls)	Diagnosis Prognosis	Fatty acid transport through the mitochondrial membrane via the carnitine shuttle. Long-chain fatty acids important for tissues and enriched in hypoxic tissues. Role in beta-oxidation.
Asparagine [87]	Non-essential amino acid	Diagnosis	Amino donor in urea, pyrimidine and purine synthesis. Supports protein synthesis during glutamine starvation. Also found in CVF fluids.
Bile acids [71]	Steroid acids	Diagnosis Prognosis (Recurrence after surgery)	Increase myometrial sensitivity to hormones, have pro-inflammatory properties and modulate cholesterol homeostasis. Act with steroids to promote EC growth, involved in signalling.
Bradykinin [71]	Polypeptide	Diagnosis Prognosis (Elevated in Type 1 EC)	Promotes inflammation, a vasodilator. Causes the release of prostacyclin and nitric oxide. Activates phospholipase D. Triggers kinin-activated pathways.
Ceramides [71,113]	Lipids	Diagnosis Prognosis (Linked to Type 2 EC recurrence)	Composed of sphingosine and a fatty acid. Involved in cell signalling, differentiation, proliferation and programmed cell death.
Cholines/acylcholines [71]	Conjugated lipids	Diagnosis Prognosis (Elevated in Type 2 EC)	Choline is necessary for the production of acetylcholine, a neurotransmitter and S-adenosyl methionine, a methyl donor in homocysteine synthesis. Acylcholines enhance penetration of estradiol in tissues. Also found in tissues and CVF fluids [79].
Cystathionine [71]	Modified amino acid	Diagnosis	Intermediate in the synthesis of cysteine. Product of homocysteine.
Estrogen metabolites [99,100,101]	Hormone	Diagnosis Prognosis	Modulates growth of the endometrium by inducing proliferation.
Glycine [71]	Amino acid	Prognosis (Elevated in Type 2 EC)	Proteinogenic amino acid. Integral to the formation of alpha-helices in secondary protein structure. Inhibitory neurotransmitter.
Heme [71]	Iron-containing porphyrin	Diagnosis Prognosis (Elevated in Type 2 EC)	A viable source of electrons during electron transfer. Modifications in Heme synthesis related pathways such as tetra-hydrofolate serine glycine pathway implicated in EC.
Hexadecadienyl carnitine/phosphatidylcholine with diacyl residue C38:1 [98]	Carnitine/choline	Prognosis (LVSI)	Carnitine-phosphatidylcholine ratio shown to be associated with presence/absence of LVSI.
Hexadecanoylcarnitine/phosphatidylcholine with acyl-alkyl residue C40:1 [98]	Carnitine/choline	Diagnosis/early detection	Carnitine-phosphatidylcholine ratio with potential for EC detection.
Homocysteine [93]	Amino acid	Diagnosis/detection Prognosis	Homologue of cysteine, a product of methionine. Sensitivity of DNA. High levels correlate with increased risk of malignant epithelial tumours.
Hydroxypropionyl carnitine [98]	Carnitine	Prognosis (Survival)	Fatty acid transport through the mitochondrial membrane via the carnitine shuttle. Long-chain fatty acids important fuels for tissues.
Hydroxysphingomyelins C14:1/hydroxysphingomyelins C24:1 [98]	Sphingomyelins	Prognosis (Myometrial invasion)	Sphingomyelin is involved in signal transduction. Degradation leads to the production of ceramide/ is involved in the apoptotic signalling pathway.
Indoleacetic acid [92]	Indoles	Diagnosis/early detection	Involved in cell proliferation/division, migration, invasion and autophagy.
Isoleucine [87]	Essential amino acid	Diagnosis	Alpha-amino acid useful in the biosynthesis of proteins. Associated with insulin resistance. Both glucogenic and ketogenic. Also found in CVF fluids [79].
Isovalerate [71]	Fatty acid	Diagnosis /early detection	Salt of isovaleric acid. Also known as 3-methyl butanoate.
Lactic acid [93]	Organic acid (Alpha-hydroxy acid)	Diagnosis Prognosis	Synthetic intermediate in metabolic pathways. Produced by pyruvate when the rate of demand for energy is high. Warburg effect. Low pH suppresses T function, promotes angiogenesis. Increases interleukin-8.
Linoleic acid [71,72,93]	Essential fatty acid	Diagnosis (Lower levels in EC) Prognosis	Unclear role in tumorigenesis. Promotes growth of mammary tumours in rodent models.
Lyso-platelet-activating factor [92]	Phospholipid	Diagnosis/early detection	Induced lipid mediator. Potent phospholipid activator and mediator of inflammation, platelet aggregation and leukocyte functions. Linked to skin cancer.
Methionine sulfoxide [111]	Essential amino acid	Prognosis (survival)	Methionine is a precursor for succinyl-CoA, homocysteine, cysteine, creatine and carnitine. Met-SO is an oxidised form of methionine.
Monoacylglycerols [71]	Glyceride	Diagnosis Prognosis	Glycerols linked to fatty acid. Act primarily as surfactants. Favour estrogenic environment.
Myristic acid [71,93]	Free fatty acid	Diagnosis (Lower levels in EC) Prognosis	Saturated fatty acids are strongly related to cholesterol concentrations. Correlate with rising triglycerides in plasma.
Phenylalanine [71,87]	Essential amino acid	Diagnosis/early detection	Precursor for tyrosine, dopamine and norepinephrine. Inhibits proliferation without affecting apoptosis or autophagy. Also found in CVF fluids [79]
Phosphatidylcholine with diacyl C42:0/phosphatidylcholine with acyl-alkyl C44:5 [98]	Lipid-like (Choline derivatives)	Diagnosis/early detection	Specific choline derivative ratios shown to predict EC.
Phosphatidylcholine with diacyl residue sum C34:4/phosphatidylcholine with acyl-alkyl C38:3 [98]	Lipid-like (Choline derivatives)	Prognosis (LVSI)	Specific choline derivative ratios are associated with presence/ absence of LVSI.
Phosphatidylcholine with diacyl residueC40:2/Phosphatidylcholine with diacyl residue C42:6 [98]	Choline derivatives	Prognosis	Specific choline derivative ratios are associated with myometrial invasion.
Phosphocholine [92]	Phospholipid	Diagnosis/early detection Prognosis	Plays a role in biosynthesis of cell membranes. Surrogate marker for cell proliferation, inhibition of invasion and migration. Protects against TNF-induced apoptosis. Also found in CVF fluids [79].
Progesterone [93]	Hormone	Diagnosis	Anti-estrogenic effect and associated with estrogen sensitivity of ECs.
Proline/tyrosine [98]	Amino acids	Diagnosis/early detection	Involved in the biosynthesis of proteins.
Sarcosine [71]	Biogenic amine	Prognosis (Elevated in Type 2 EC)	Intermediate in the metabolism of choline to glycine.
Spermine [71]	Biogenic amine	Diagnosis/early detection Prognosis	Likely originating from EC cells. Involved in cellular metabolism.
Sphingolipids [71]	Sphingolipids	Diagnosis Prognosis	Fatty acid derivatives of sphingosine which occur in cell membranes, especially of the brain and nervous tissues. Also found in EC tissues [113].
Stearic acid [72,93]	Fatty acid	Diagnosis/early detection	Saturated fatty acid with surfactant properties. In vitro inhibition of cancer cell growth. Downregulated in EC.
Sulfated androgens [71]	Sulfated androgens	Diagnosis Prognosis	Sulfated androgens implicated in Type 1 EC. Role in sexual development of males.
Tetradecadienoylcarnitine [77]	Carnitine	Diagnosis/early detection	Energy metabolism and fatty acid transport.
Threonine [93]	Amino acid	Diagnosis/early detection	Amino acid involved in protein biosynthesis.
Valine [93]	Amino acid	Diagnosis	An amino acid used in the biosynthesis of proteins.

### 4.2. Tissue-Based Metabolomic Biomarkers

Biomarkers measured in blood, urine or other minimally invasive samples are ideal for diagnostic and recurrence monitoring purposes. Cancer-specific biomarkers are likely to be more successful than those linked to risk factors, such as obesity or the ageing process, however these may be difficult to distinguish, particularly if cancer-relevant biomarkers are in relatively low abundance in biofluids distant to the tumour. To circumnavigate this issue, some researchers have discovered metabolomic biomarkers in tumour tissue with a view to their subsequent identification, albeit at much lower concentrations, in distant biofluids and blood circulation (Table 2). Altadil and colleagues found glycerophospholipids to be upregulated in EC tissues, specifically glycerophosphocholines (PCs), phosphatidylserine (PSs), phosphatidylethanolamines (PEs), phosphatidylinositols (PIs) and phosphatidylglycerol (PGs) [80]. These findings are consistent with those by Trousil and colleagues who also reported an increase of up to 70% in phosphocholine levels of EC tissues in a study comparing endometrial tissues from 10 high-grade endometrioid EC cases and 10 benign controls [78]. Phosphocholines play crucial roles in cell membrane synthesis and are thus surrogates for cell proliferation, a cardinal feature of tumorigenesis. They have also been reported in blood [93] and cervicovaginal fluid samples [79], suggesting that they may be viable candidates for EC detection. Other metabolic alterations in EC tissues included the downregulation of palmitamide, stearamide, oleamide, glutamine/tryptophan and inosine and upregulation of 3-Deoxyvitamin D3, linoleic acid, UDP-N-acetyl-d-galactosamine, sphingaline, sphingosine and dihydroceramide, among others [80,113,114].

Eritja and colleagues, studied the tumour microenvironmental blood flow of EC using dynamic contrast-enhanced magnetic resonance imaging alongside liquid chromatography coupled to mass spectrometry and reported lysophospholipids and resolvin D as EC metabolic biomarkers [115]. Lysophospholipids are important constituents of cell membranes and have been postulated to exhibit pro-angiogenic and anti-apoptotic properties [116]. Resolvin D, on the other hand, has been implicated in the resolution of inflammatory processes [117]. These markers are yet to be externally validated and further studies are needed.

Prognostic metabolomic biomarkers identified in tumour tissue include picolinic acid, vaccenic acid, phosphatidic acid, arachidonic acid, 13*Z*-docosenamide, UDP-*N*-acetyl-d-galactosamine, 1-palmitoyl-2-linoleoyl, inosine, palmitic amide, gleamide, linoleic acid, phosphatidylserine, phosphatidylinositol and glycerophosphocholines, among others [76,80] (Table 1). Picolinic acid is an end-product of the kynurenine pathway which is downregulated in EC, in keeping with its reported anti-tumoral properties, while UDP-N-acetyl-D-galactosamine and arachidonic acid are upregulated in advanced stages of EC [80].

**Table 2:** Most promising tissue-based EC metabolomic biomarker candidates and their functions.

Metabolite	Group/Sub-Class	Potential Clinical Utility	Biochemical Function and Summary of Evidence
13Z- Docosenamide [80]	Primary fatty amide	Diagnosis Prognosis	An amide of docosenoic acid. Unclear mechanism relating to EC development and progression.
1-palmitoyl-2-linoleoyl-glycero-3phosphocholine [80]	Diacylglycerol and phospholipid	Diagnosis Prognosis	Component of biological membranes. Involved in membrane-mediated cell signalling.
5,8,11-eicosatrienoic acid [80]	Straight chain fatty acid	Diagnosis Prognosis	Belong to eicosanoids, synthesised from oxidised polyunsaturated fatty acids, mediate cell–cell communication and inflammatory immune response.
Arachidonic acid [80]	Polyunsaturated fatty acid	Diagnosis Prognosis	Present in phospholipids of membranes, plays roles in the synthesis of prostaglandins and leukotrienes.
Capric acid [118]	Saturated fatty acid	Diagnosis	Downregulated in EC. Role in cell signaling, energy storage, membrane stability. In vitro inhibition of cancer proliferation.
Cholines/acylcholines [78,80]	Conjugated lipids	Diagnosis Prognosis (Elevated in Type 2 EC)	Acylcholines enhance penetration of estradiol in tissues. Seen in blood [71] and CVF [79].
Glutamate/arginine/Tryptophan [80]	Amino acids	Diagnosis Prognosis	Bio-active amino acids. Metabolic fuels. Also reported in plasma [87].
Glycerophosphocholines [78,80]	Natural choline	Diagnosis Prognosis	Biosynthetic precursors of acetylcholine. Up to 70% increase in EC tissues.
Hypoxanthine [80]	Purine metabolite	Prognosis (myometrial invasion)	Purine derivative, a constituent of nucleic acids present in the anticodon of tRNA.
Inosine [78,80]	Purine metabolite	Diagnosis Prognosis	Nucleoside found in tRNAs and essential for translation of the genetic code in wobble base pairs. Imbalance in isoleucine–alanine ratio.
Monoacylglycerol [118]	Acylglycerol	Diagnosis	Monoacylglycerol 24:0 significantly downregulated in EC tissues. Modulates cellular processes including proliferation and apoptosis.
Oleamide [80]	Fatty acid amide	Diagnosis Prognosis (Increased in grade 3 EC)	Mechanism of action is unclear. Modulator of neurotransmitter and voltage-gated ion channel activity.
Palmitic amide [80]	Amide	Diagnosis Prognosis	Primary fatty acid amide.
Phosphatidic acid [80,115]	Phospholipids	Diagnosis Prognosis	Anionic phospholipids important in cell signalling and activation of lipid-gated ion channels.
Phosphatidylethanolamines [80]	Phospholipids	Diagnosis Prognosis	Phospholipids found in biological membranes. Involved in membrane fusion and cytokinesis/cell division. Regulate membrane curvature.
Phosphatidylglycerol [80]	Phospholipids	Diagnosis Prognosis	Glycerophospholipid and pulmonary surfactant. Activates lipid-gated ion channels.
Phosphatidylinositols [80]	Phospholipids	Diagnosis Prognosis	Acidic phospholipids involved in lipid signalling, cell signalling and membrane trafficking.
Phosphatidylserine [80]	Phospholipids	Diagnosis Prognosis	Role in cell signalling, especially in brain cells.
Picolinic acid [80]	Pyridine derivative	Diagnosis Prognosis	Catabolite of tryptophan through the kynurenine pathway. Unclear function. Possible immunological and anti-proliferative/ anti-tumoral effects.
Sphingolipids [113]	Sphingolipid	Diagnosis Prognosis	Fatty acid derivatives of sphingosine. Also reported in blood [71].
Stearamide [80]	Endocannabinoid	Diagnosis Prognosis	Endocannabinoids regulate cell proliferation, differentiation and cell survival.
Taurine [80]	Amino sulfonic acid	Prognosis (Type 1 EC)	Amino sulfonic acids, naturally occurring, found in muscles, brain, eyes and heart. Decreased in high-grade EC.
UDP-*N*-acetyl-d–galactosamine [80]	Hexosamine	Diagnosis Prognosis	Linked to the metabolism of glucose, fatty acids, and amino acids.
Vaccenic acid [80]	Fatty acid	Diagnosis Prognosis	Trans fatty acid which in mammals is converted into rumenic acid, where it shows anti-carcinogenic properties.
Xanthine [80]	Purine metabolite	Prognosis (Myometrial invasion)	Product of purine degradation, created from guanine by the actions of guanine deaminase.

### 4.3. Urine Based Metabolomics Biomarkers

Urine has several advantages as a source of diagnostic markers as it is non-invasive and easy to collect with no associated harms [67]. Urinary metabolites may originate from systemic metabolites that are chemically modified and excreted in urine, or from contamination of urine by tumour-derived metabolites shed into the lower genital tract [119]. Only a few urinary metabolomic studies have been conducted in the context of EC (Table 3). Shao and colleagues subjected urinary samples from 25 EC cases, 25 healthy controls and 10 endometrial hyperplasia (EH) controls to liquid chromatography quadrupole time-of-flight mass spectrometry (LC-Q-TOF/MS) and reported lower levels of acetylcysteine and porphobilinogen in EC cases and higher levels of N-acetylserine, urocanic acid and isobutyrylglycine [120]. A five-panel diagnostic algorithm was reported as being able to distinguish EC cases from the merged group of EH and healthy controls [120]. These markers are yet to be externally validated and further studies are needed to determine their role in EC development and progression. In another study, attenuated total reflection Fourier-transform infrared (ATR-FTIR) spectroscopy was used to develop a biomarker algorithm with 95% sensitivity and 100% specificity for EC detection based on an analysis of urinary specimens from 10 EC cases, 10 ovarian cancer cases and 10 healthy controls. This study was limited by the small sample size and the algorithm is yet to be externally validated [47]. A number of metabolites have shown potential for the discrimination of EC from benign ovarian tumours and include: S-reticuline, n-acetylneuraminic acid, 3-sialyl-n-acetyllactosamine, 3-dehydroquinic acid, 3-indoleacetic acid, selenocystathionine, 1-(1Z-hexadecenyl)-sn-glycero-3-3-phosphate and 3-sialylactose [46,67].

Urinary endogenous estrogen metabolites have also been explored as potential EC biomarkers. Using samples from 23 EC cases and 23 healthy controls, Zhao and colleagues reported 4-hydroxyestradiol (4-OHE2) and 17β-estradiol (E2) to be upregulated in EC, while 2-methoxyestrone (2-MeOE1) and 2-methoxyestradiol (2-MeOE2) were downregulated [121]. 17β-estradiol E2 has been implicated in the activation of P13K/AKT and MAPK signalling pathways, both of which are known to be dysregulated in EC. 4-hydroxyestradiol upregulates CYP1B1, a member of the cytochrome family of enzymes which is known to enhance cell proliferation and metastasis, while 2-methoxyestrone has anti-proliferative and pro-apoptotic properties, consistent with its downregulation in EC [121].

Whilst metabolomic analysis of urine has the potential to deliver clinically relevant biomarkers, this is only possible when environmental conditions are controlled to avoid introducing spurious signals that lead to false positive findings (Figure 2). As an example, contamination of self-collected urine samples by commensal bacteria of the lower genital tract can affect the concentrations of metabolites in urine [122]. Bacterial overgrowth may occur under room temperature storage conditions, leading to modification of urine composition following the consumption and production of new metabolites [46,122].

### 4.4. EC Detection in Minimally Invasive Genital Samples

Given the anatomical continuity of the uterus and lower genital tract, there is growing interest in the potential of developing EC biomarkers based on minimally invasive sampling methodologies [45]. Cervicovaginal fluids (CVF), for example, contain cervical and endometrial gland secretions and are viable sources of EC-derived metabolites [79]. There are multiple reports on the utility of CVF for the detection of inflammatory and malignant conditions of the lower genital tract, including cervical cancer and bacterial vaginosis [45,123,124]. Cheng and colleagues used nuclear magnetic resonance spectroscopy to compare the CVF metabolomic profile of 21 EC cases and 33 non-EC controls and reported choline, formate, fumarate, phosphocholine and malate to be overexpressed in EC [79]. In contrast, phenylalanine, aspartate, asparagine, isoleucine and pyruvate were significantly downregulated. Of these biomarker candidates, phosphocholine had the best diagnostic performance, with an AUC of 0.82 (95% CI 0.69–0.93), while the model based on phosphocholine, malate and asparagine had potential to not only predict EC but also other gynaecological malignancies [79]. Phosphocholine, a surrogate for cell proliferation, has been linked to high-grade EC [125]. Table 4 summarises the biochemical functions of CVF metabolites, however, these markers are yet to be externally validated and larger studies using more sensitive metabolomic platforms are needed to explore the feasibility of CVF-derived metabolites as biomarkers of EC.

## 5. Conclusions

In this review, we have described the current status of EC metabolomic biomarker research and underscored the challenges inherent in biomarker discovery and validation. Lipid, amino acid and hormonal metabolites have all been reported as potential EC biomarkers for detection, prognosis and monitoring for disease recurrence. Whilst the evidence to enable translation into clinical practice is lacking, the results are encouraging and further studies are needed to validate the identified markers and to elucidate their role in EC tumorigenesis. When used alongside minimally invasive sampling methodologies, metabolomics has enormous potential to deliver clinically relevant biomarkers that can be translated into routine clinical practice. Future metabolomic studies should aim to identify metabolites linked to the TCGA EC molecular subtypes, which offer better prognostic discrimination than traditional EC histological subtypes. Advances in the use of artificial intelligence and machine learning techniques to combine metabolic signals from multiple studies have potential to enable the generation of a robust metabolomic biomarker panel for EC detection.

## Figures and Tables

**Table 3:** Most promising urine-based EC metabolomic biomarker candidates and their functions.

Metabolite	Group/Sub-Class	Potential Clinical Utility	Biochemical Function and Summary of Evidence
Acetylcysteine [120]	Amino acid metabolite	Diagnosis	Precursor of the anti-oxidant glutathione. Able to reduce free radicals. Found to be downregulated in EC.
Estrogens [121]	Hormones	Diagnosis	Female sex hormones, endometrial proliferation. 4-hydroxyestrone found to be elevated in EC. 2-methoxyestrone and 2-methoxyestradiol were downregulated in EC.
Isobutyrylglycine [120]	Acyl glycine	Diagnosis	Minor metabolite of fatty acids and known urinary metabolite. A conjugate acid of N-isobutyrylglycinate. Found to be upregulated in EC.
N-acetylserine [120]	Amino acid	Diagnosis	Acetylation of the serine amino acid N-terminal. Found to be upregulated in EC.
Porphobilinogen [120]	Amine	Diagnosis	Pyrrole intermediate in the synthesis of porphyrin. Found to be downregulated in EC.
Urocanic acid [120]	Deamination product	Diagnosis	Breakdown product of histidine. Found to be upregulated in EC.

**Table 4:** Most promising cervicovaginal fluid-based EC metabolomic biomarker candidates and their functions.

Metabolite	Group/Sub-Class	Potential Clinical Utility	Biochemical Function and Summary of Evidence
Fumarate [79]	Organic acid (Dicarboxylate)	Diagnosis/early detection	Intermediate in the citric acid cycle. Converted to malate. Citric cycle releases stored energy through the oxidation of acetyl-CoA.
Malate [79]	Dicarboxylic acid	Diagnosis/early detection	Intermediate in the citric acid cycle
Isoleucine [79]	Essential amino acid	Diagnosis	Alpha-amino acid useful in the biosynthesis of proteins. Associated with insulin resistance. Both glucogenic and ketogenic. Reported in serum [87].
Asparagine [79]	Non-essential amino acid	Diagnosis/early detection	Amino donor in urea, pyrimidine and purine synthesis. Supports protein synthesis during glutamine starvation. Reported in serum [87].
Aspartate [79]	Non-essential amino acid	Diagnosis	Involved in protein synthesis and neurotransmission.
Cholines/acylcholines [79]	Conjugated lipids	Diagnosis Prognosis (elevated in Type 2 EC)	Necessary for homocysteine synthesis. Acylcholines enhance penetration of estradiol in tissues. Reported in tissue/serum [78,80].
Phenylalanine [79]	Essential amino acid	Diagnosis Early detection	Precursor for tyrosine, dopamine and norepinephrine. Inhibits proliferation without affecting apoptosis or autophagy. Also reported in plasma [71,92].
Phosphocholine [79,92]	Phospholipid	Diagnosis Prognosis (high-grade EC)	Plays a role in biosynthesis of cell membranes. Surrogate marker for cell proliferation, inhibition of invasion and migration. Protects against TNF-induced apoptosis. Elevated in CVF of EC patients. Also seen in plasma [92].

## References

[1] Ferlay J., Colombet M., Soerjomataram I., Mathers C., Parkin D.M., Piñeros M., Znaor A., Bray F. (2019). Estimating the Global Cancer Incidence and Mortality in 2018: GLOBOCAN Sources and Methods. Int. J. Cancer.

[2] Bray F., Ferlay J., Soerjomataram I., Siegel R.L., Torre L.A., Jemal A. (2018). Global Cancer Statistics 2018: GLOBOCAN Estimates of Incidence and Mortality Worldwide for 36 Cancers in 185 Countries. CA Cancer J. Clin..

[3] Crosbie E., Morrison J. (2014). The Emerging Epidemic of Endometrial Cancer: Time to Take Action. Cochrane Database Syst. Rev..

[4] CRUK (2020). Uterine Cancer Incidence Statistics. www.Cancerresearchuk.Org.

[5] Sundar S., Balega J., Crosbie E., Drake A., Edmondson R., Fotopoulou C., Gallos I., Ganesan R., Gupta J., Johnson N. (2017). BGCS Uterine Cancer Guidelines: Recommendations for Practice. Eur. J. Obstet. Gynecol. Reprod. Biol..

[6] WHO Obesity and Overweight. https://www.who.int/news-room/fact-sheets/detail/obesity-and-overweight.

[7] Hales C.M., Carroll M.D., Fryar C.D., Ogden C.L. Prevalence of Obesity and Severe Obesity among Adults: United States, 2017–2018. NCHS Data Brief No 360, February 2020. https://www.cdc.gov/nchs/products/databriefs/db360.htm.

[8] Gynaecologists (2012). Royal College of Obstetricians and Gynaecologists (RCOG) Scientific Impact Paper No. 32 on Endometrial Cancer in Obese Women. https://www.rcog.org.uk/globalassets/documents/guidelines/scientific-impact-papers/sip_32.pdf.

[9] Colombo N., Creutzberg C., Amant F., Bosse T., González-Martín A., Ledermann J., Marth C., Nout R., Querleu D., Mirza M.R. (2016). ESMO-ESGO-ESTRO Consensus Conference on Endometrial Cancer: Diagnosis, Treatment and Follow-Up. Int. J. Gynecol. Cancer.

[10] Vandecaveye V. (2020). Advances in Endometrial Cancer Diagnosis. Management of Endometrial Cancer.

[11] Casadio P., Magnarelli G., Alletto A., Guasina F., Morra C., Talamo M.R., La Rosa M., Su H., Frisoni J., Seracchioli R. (2020). Endometrial Cancer. Atlas of Hysteroscopy.

[12] Clark T.J., Voit D., Gupta J.K., Hyde C., Song F., Khan K.S. (2002). Accuracy of Hysteroscopy in the Diagnosis of Endometrial Cancer and Hyperplasia: A Systematic Quantitative Review. JAMA.

[13] Njoku K., Chiasserini D., Whetton A.D., Crosbie E.J. (2019). Proteomic Biomarkers for the Detection of Endometrial Cancer. Cancers.

[14] Rižner T.L. (2016). Discovery of Biomarkers for Endometrial Cancer: Current Status and Prospects. Expert Rev. Mol. Diagn..

[15] Badrick E., Cresswell K., Ellis P., Crosbie P., Hall P.S., O’Flynn H., Martin R., Leighton J., Brown L., Makin D. (2019). Top Ten Research Priorities for Detecting Cancer Early. Lancet Public Health.

[16] Bokhman J.V. (1983). Two Pathogenetic Types of Endometrial Carcinoma. Gynecol. Oncol..

[17] Ceppi L., Dizon D.S., Birrer M.J. (2020). Endometrial Cancer Genetic Classification and Its Clinical Application. Management of Endometrial Cancer.

[18] Levine D.A., Cancer Genome Atlas Research Network (2013). Integrated Genomic Characterization of Endometrial Carcinoma. Nature.

[19] Talhouk A., McConechy M.K., Leung S., Yang W., Lum A., Senz J., Boyd N., Pike J., Anglesio M., Kwon J.S. (2017). Confirmation of ProMisE: A Simple, Genomics-based Clinical Classifier for Endometrial Cancer. Cancer.

[20] Stelloo E., Bosse T., Nout R.A., MacKay H.J., Church D.N., Nijman H.W., Leary A., Edmondson R.J., Powell M.E., Crosbie E.J. (2015). Refining Prognosis and Identifying Targetable Pathways for High-Risk Endometrial Cancer; a TransPORTEC Initiative. Mod. Pathol..

[21] Yen T.-T., Wang T.-L., Fader A.N., Shih I.-M., Gaillard S. (2020). Molecular Classification and Emerging Targeted Therapy in Endometrial Cancer. Int. J. Gynecol. Pathol..

[22] Vermij L., Smit V., Nout R., Bosse T. (2020). Incorporation of Molecular Characteristics into Endometrial Cancer Management. Histopathology.

[23] Njoku K., Abiola J., Russell J., Crosbie E.J. (2020). Endometrial Cancer Prevention in High-Risk Women. Best Pract. Res. Clin. Obstet. Gynaecol..

[24] Wan Y.L., Beverley-Stevenson R., Carlisle D., Clarke S., Edmondson R.J., Glover S., Holland J., Hughes C., Kitchener H.C., Kitson S. (2016). Working Together to Shape the Endometrial Cancer Research Agenda: The Top Ten Unanswered Research Questions. Gynecol. Oncol..

[25] Hardiman G. (2020). An Introduction to Systems Analytics and Integration of Big Omics Data. Genes.

[26] Lockhart D.J., Winzeler E.A. (2000). Genomics, Gene Expression and DNA Arrays. Nature.

[27] Hasin Y., Seldin M., Lusis A. (2017). Multi-Omics Approaches to Disease. Genome Biol..

[28] Jacob M., Lopata A.L., Dasouki M., Abdel Rahman A.M. (2019). Metabolomics toward Personalized Medicine. Mass Spectrom. Rev..

[29] Wishart D.S., Mandal R., Stanislaus A., Ramirez-Gaona M. (2016). Cancer Metabolomics and the Human Metabolome Database. Metabolites.

[30] Yang X., Wang J. (2019). The Role of Metabolic Syndrome in Endometrial Cancer: A Review. Front. Oncol..

[31] Altadill T. Metabolomic Pathway Alterations in Endometrial Cancer. Proceedings of the AACR Annual Meeting.

[32] Crosbie E.J., Zwahlen M., Kitchener H.C., Egger M., Renehan A.G. (2010). Body Mass Index, Hormone Replacement Therapy, and Endometrial Cancer Risk: A Meta-Analysis. Cancer Epidemiol. Prev. Biomark..

[33] Perry R.J., Shulman G.I. (2020). Mechanistic Links between Obesity, Insulin, and Cancer. Trends Cancer.

[34] Kitson S.J., Evans D.G., Crosbie E.J. (2017). Identifying High-Risk Women for Endometrial Cancer Prevention Strategies: Proposal of an Endometrial Cancer Risk Prediction Model. Cancer Prev. Res..

[35] Cai Y., Chang Y., Liu Y. (2019). Multi-Omics Profiling Reveals Distinct Microenvironment Characterization of Endometrial Cancer. Biomed. Pharmacother..

[36] Chen P., Yang Y., Zhang Y., Jiang S., Li X., Wan J. (2020). Identification of Prognostic Immune-Related Genes in the Tumor Microenvironment of Endometrial Cancer. Aging.

[37] Giannone G., Attademo L., Scotto G., Genta S., Ghisoni E., Tuninetti V., Aglietta M., Pignata S., Valabrega G. (2019). Endometrial Cancer Stem Cells: Role, Characterization and Therapeutic Implications. Cancers.

[38] Felix A., Weissfeld J., Edwards R., Linkov F. (2010). Future Directions in the Field of Endometrial Cancer Research: The Need to Investigate the Tumor Microenvironment. Eur. J. Gynaecol. Oncol..

[39] Pinu F.R., Beale D.J., Paten A.M., Kouremenos K., Swarup S., Schirra H.J., Wishart D. (2019). Systems Biology and Multi-Omics Integration: Viewpoints from the Metabolomics Research Community. Metabolites.

[40] Gomez-Casati D.F., Busi M.V. (2020). Molecular Basis of Clinical Metabolomics. Clinical Molecular Medicine.

[41] Wishart D.S., Feunang Y.D., Marcu A., Guo A.C., Liang K., Vázquez-Fresno R., Sajed T., Johnson D., Li C., Karu N. (2018). HMDB 4.0: The Human Metabolome Database for 2018. Nucleic Acids Res..

[42] Kohler I., Hankemeier T., van der Graaf P.H., Knibbe C.A.J., van Hasselt J.G.C. (2017). Integrating Clinical Metabolomics-Based Biomarker Discovery and Clinical Pharmacology to Enable Precision Medicine. Eur. J. Pharm. Sci..

[43] Armitage E.G., Barbas C. (2014). Metabolomics in Cancer Biomarker Discovery: Current Trends and Future Perspectives. J. Pharm. Biomed. Anal..

[44] Basetti M. (2017). Cancer Metabolism. Metabolites.

[45] Costas L., Frias-Gomez J., Guardiola M., Benavente Y., Pineda M., Pavón M.Á., Martínez J.M., Climent M., Barahona M., Canet J. (2019). New Perspectives on Screening and Early Detection of Endometrial Cancer. Int. J. Cancer.

[46] Zhang A., Sun H., Wu X., Wang X. (2012). Urine Metabolomics. Clin. Chim. Acta.

[47] Paraskevaidi M., Morais C.L.M., Lima K.M.G., Ashton K.M., Stringfellow H.F., Martin-Hirsch P.L., Martin F.L. (2018). Potential of Mid-Infrared Spectroscopy as a Non-Invasive Diagnostic Test in Urine for Endometrial or Ovarian Cancer. Analyst.

[48] Bingol K. (2018). Recent Advances in Targeted and Untargeted Metabolomics by NMR and MS/NMR Methods. High-Throughput.

[49] Tolstikov V., Moser A.J., Sarangarajan R., Narain N.R., Kiebish M.A. (2020). Current Status of Metabolomic Biomarker Discovery: Impact of Study Design and Demographic Characteristics. Metabolites.

[50] Le A., Mak J., Cowan T.M. (2020). Metabolic Profiling by Reversed-Phase/Ion-Exchange Mass Spectrometry. J. Chromatogr. B.

[51] Damiani C., Gaglio D., Sacco E., Alberghina L., Vanoni M. (2020). Systems Metabolomics: From Metabolomic Snapshots to Design Principles. Curr. Opin. Biotechnol..

[52] Bracewell-Milnes T., Saso S., Abdalla H., Nikolau D., Norman-Taylor J., Johnson M., Holmes E., Thum M.-Y. (2017). Metabolomics as a Tool to Identify Biomarkers to Predict and Improve Outcomes in Reproductive Medicine: A Systematic Review. Hum. Reprod. Update.

[53] Banach P., Suchy W., Dereziński P., Matysiak J., Kokot Z.J., Nowak-Markwitz E. (2017). Mass Spectrometry as a Tool for Biomarkers Searching in Gynecological Oncology. Biomed. Pharmacother..

[54] Djukovic D., Raftery D., Gowda N. (2020). Mass Spectrometry and NMR Spectroscopy Based Quantitative Metabolomics. Proteomic and Metabolomic Approaches to Biomarker Discovery.

[55] Theodoridis G.A., Gika H.G., Plumb R., Wilson I.D. (2020). Liquid Chromatographic Methods Combined with Mass Spectrometry in Metabolomics. Proteomic and Metabolomic Approaches to Biomarker Discovery.

[56] López-Gonzálvez Á., Godzien J., García A., Barbas C. (2019). Capillary Electrophoresis Mass Spectrometry as a Tool for Untargeted Metabolomics. High-Throughput Metabolomics.

[57] Wishart D.S. (2019). NMR Metabolomics: A Look Ahead. J. Magn. Reson..

[58] Cameron J.M., Bruno C., Parachalil D.R., Baker M.J., Bonnier F., Butler H.J., Byrne H.J. (2020). Vibrational Spectroscopic Analysis and Quantification of Proteins in Human Blood Plasma and Serum. Vibrational Spectroscopy in Protein Research.

[59] Perez E.A. (2020). Biomarkers and Precision Medicine in Oncology Practice and Clinical Trials. Advancing the Science of Cancer in Latinos.

[60] Chang J.Y.H., Ladame S. (2020). Diagnostic, Prognostic, and Predictive Biomarkers for Cancer. Bioengineering Innovative Solutions for Cancer.

[61] Page M.J. (2020). Confounding and Other Concerns in Meta-Epidemiological Studies of Bias. J. Clin. Epidemiol..

[62] McKeigue P. (2019). Sample Size Requirements for Learning to Classify with High-Dimensional Biomarker Panels. Stat. Methods Med. Res..

[63] Dunn W.B., Lin W., Broadhurst D., Begley P., Brown M., Zelená E., Vaughan A.A., Halsall A., Harding N., Knowles J. (2015). Molecular phenotyping of a UK population: Defining the human serum metabolome. Metabolomics.

[64] González-Domínguez R., González-Domínguez Á., Sayago A., Fernández-Recamales Á. (2020). Recommendations and Best Practices for Standardizing the Pre-Analytical Processing of Blood and Urine Samples in Metabolomics. Metabolites.

[65] Lucas-Torres C., Bernard T., Huber G., Berthault P., Nishiyama Y., Kandiyal P.S., Elena-Herrmann B., Molin L., Solari F., Bouzier-Sore A.-K. (2020). General Guidelines for Sample Preparation Strategies in HR-ΜMAS NMR-Based Metabolomics of Microscopic Specimens. Metabolites.

[66] Rinschen M.M., Ivanisevic J., Giera M., Siuzdak G. (2019). Identification of Bioactive Metabolites Using Activity Metabolomics. Nat. Rev. Mol. Cell Biol..

[67] Dinges S.S., Hohm A., Vandergrift L.A., Nowak J., Habbel P., Kaltashov I.A., Cheng L.L. (2019). Cancer Metabolomic Markers in Urine: Evidence, Techniques and Recommendations. Nat. Rev. Urol..

[68] Jolliffe I.T., Cadima J. (2016). Principal Component Analysis: A Review and Recent Developments. Philos. Trans. R. Soc. Math. Phys. Eng. Sci..

[69] Lee L.C., Liong C.-Y., Jemain A.A. (2018). Partial Least Squares-Discriminant Analysis (PLS-DA) for Classification of High-Dimensional (HD) Data: A Review of Contemporary Practice Strategies and Knowledge Gaps. Analyst.

[70] Ueda Y., Enomoto T., Kimura T., Miyatake T., Yoshino K., Fujita M., Kimura T. (2010). Serum Biomarkers for Early Detection of Gynecologic Cancers. Cancers.

[71] Audet-Delage Y., Villeneuve L., Grégoire J., Plante M., Guillemette C. (2018). Identification of Metabolomic Biomarkers for Endometrial Cancer and Its Recurrence after Surgery in Postmenopausal Women. Front. Endocrinol..

[72] Gaudet M.M., Falk R.T., Stevens R.D., Gunter M.J., Bain J.R., Pfeiffer R.M., Potischman N., Lissowska J., Peplonska B., Brinton L.A. (2012). Analysis of Serum Metabolic Profiles in Women with Endometrial Cancer and Controls in a Population-Based Case-Control Study. J. Clin. Endocrinol. Metab..

[73] Strimbu K., Tavel J.A. (2010). What Are Biomarkers?. Curr. Opin. HIV AIDS.

[74] Engerud H.R. (2020). Molecular Markers to Predict Prognosis and Guide Therapy in Endometrial Cancer. Ph.D. Thesis.

[75] Gentry-Maharaj A., Karpinskyj C. (2020). Current and Future Approaches to Screening for Endometrial Cancer. Best Pract. Res. Clin. Obstet. Gynaecol..

[76] Raffone A., Troisi J., Boccia D., Travaglino A., Capuano G., Insabato L., Mollo A., Guida M., Zullo F. (2020). Metabolomics in Endometrial Cancer Diagnosis: A Systematic Review. Acta Obstet. Gynecol. Scand..

[77] Bahado-Singh R.O., Lugade A., Field J., Al-Wahab Z., Han B., Mandal R., Bjorndahl T.C., Turkoglu O., Graham S.F., Wishart D. (2018). Metabolomic Prediction of Endometrial Cancer. Metabolomics.

[78] Trousil S., Lee P., Pinato D.J., Ellis J.K., Dina R., Aboagye E.O., Keun H.C., Sharma R. (2014). Alterations of Choline Phospholipid Metabolism in Endometrial Cancer Are Caused by Choline Kinase Alpha Overexpression and a Hyperactivated Deacylation Pathway. Cancer Res..

[79] Cheng S.-C., Chen K., Chiu C.-Y., Lu K.-Y., Lu H.-Y., Chiang M.-H., Tsai C.-K., Lo C.-J., Cheng M.-L., Chang T.-C. (2019). Metabolomic Biomarkers in Cervicovaginal Fluid for Detecting Endometrial Cancer through Nuclear Magnetic Resonance Spectroscopy. Metabolomics.

[80] Altadill T., Dowdy T.M., Gill K., Reques A., Menon S.S., Moiola C.P., Lopez-Gil C., Coll E., Matias-Guiu X., Cabrera S. (2017). Metabolomic and Lipidomic Profiling Identifies the Role of the RNA Editing Pathway in Endometrial Carcinogenesis. Sci. Rep..

[81] Loftsson T., Somogyi G., Bodor N. (1989). Effect of Choline Esters and Oleic Acid on the Penetration of Acyclovir, Estradiol, Hydrocortisone, Nitroglycerin, Retinoic Acid and Trifluorothymidine across Hairless Mouse Skin in Vitro. Acta Pharm. Nord..

[82] Chughtai K., Jiang L., Greenwood T.R., Glunde K., Heeren R.M.A. (2013). Mass Spectrometry Images Acylcarnitines, Phosphatidylcholines, and Sphingomyelin in MDA-MB-231 Breast Tumor Models. J. Lipid Res..

[83] Qin H., Ruan Z. (2014). The Role of Monoacylglycerol Lipase (MAGL) in the Cancer Progress. Cell Biochem. Biophys..

[84] Paraskevaidi M., Morais C.L.M., Ashton K.M., Stringfellow H.F., McVey R.J., Ryan N.A.J., O’Flynn H., Sivalingam V.N., Kitson S.J., MacKintosh M.L. (2020). Detecting Endometrial Cancer by Blood Spectroscopy: A Diagnostic Cross-Sectional Study. Cancers.

[85] O’Connell T.M. (2013). The Complex Role of Branched Chain Amino Acids in Diabetes and Cancer. Metabolites.

[86] Lieu E.L., Nguyen T., Rhyne S., Kim J. (2020). Amino Acids in Cancer. Exp. Mol. Med..

[87] Ihata Y., Miyagi E., Numazaki R., Muramatsu T., Imaizumi A., Yamamoto H., Yamakado M., Okamoto N., Hirahara F. (2014). Amino Acid Profile Index for Early Detection of Endometrial Cancer: Verification as a Novel Diagnostic Marker. Int. J. Clin. Oncol..

[88] Okamoto N. (2012). Use of “AminoIndex Technology” for Cancer Screening. Ningen Dock.

[89] Mikami H., Kimura O., Yamamoto H., Kikuchi S., Nakamura Y., Ando T., Yamakado M. (2019). A Multicentre Clinical Validation of AminoIndex Cancer Screening (AICS). Sci. Rep..

[90] Miyagi E., Numazaki R., Nakanishi T., Kataoka F., Saruki N., Ihata Y. (2012). Diagnostic Performance and Clinical Utility of Novel Gynecologic Cancer Screening Method Based on “AminoIndex Technology”. Ningen Dock.

[91] Suzuki Y., Tokinaga-Uchiyama A., Mizushima T., Maruyama Y., Mogami T., Shikata N., Ikeda A., Yamamoto H., Miyagi E. (2018). Normalization of Abnormal Plasma Amino Acid Profile-Based Indexes in Patients with Gynecological Malignant Tumors after Curative Treatment. BMC Cancer.

[92] Shi K., Wang Q., Su Y., Xuan X., Liu Y., Chen W., Qian Y., Lash G.E. (2018). Identification and Functional Analyses of Differentially Expressed Metabolites in Early Stage Endometrial Carcinoma. Cancer Sci..

[93] Troisi J., Sarno L., Landolfi A., Scala G., Martinelli P., Venturella R., Di Cello A., Zullo F., Guida M. (2018). Metabolomic Signature of Endometrial Cancer. J. Proteome Res..

[94] Warburg O., Wind F., Negelein E. (1927). The Metabolism of Tumors in the Body. J. Gen. Physiol..

[95] Narayanan S., Santhoshkumar A., Ray S., Harihar S. (2020). Reprogramming of Cancer Cell Metabolism: Warburg and Reverse Warburg Hypothesis. Cancer Cell Metabolism: A Potential Target for Cancer Therapy.

[96] Polet F., Feron O. (2013). Endothelial Cell Metabolism and Tumour Angiogenesis: Glucose and Glutamine as Essential Fuels and Lactate as the Driving Force. J. Intern. Med..

[97] Shen W., Gao C., Cueto R., Liu L., Fu H., Shao Y., Yang W.Y., Fang P., Choi E.T., Wu Q. (2020). Homocysteine-Methionine Cycle Is a Metabolic Sensor System Controlling Methylation-Regulated Pathological Signaling. Redox Biol..

[98] Knific T., Vouk K., Smrkolj Š., Prehn C., Adamski J., Rižner T.L. (2018). Models Including Plasma Levels of Sphingomyelins and Phosphatidylcholines as Diagnostic and Prognostic Biomarkers of Endometrial Cancer. J. Steroid Biochem. Mol. Biol..

[99] Brinton L.A., Trabert B., Anderson G.L., Falk R.T., Felix A.S., Fuhrman B.J., Gass M.L., Kuller L.H., Pfeiffer R.M., Rohan T.E. (2016). Serum Estrogens and Estrogen Metabolites and Endometrial Cancer Risk among Postmenopausal Women. Cancer Epidemiol. Prev. Biomark..

[100] Audet-Walsh E., Lepine J., Gregoire J., Plante M., Caron P., Teˆtu B., Ayotte P., Brisson J., Villeneuve L., Belanger A. (2011). Profiling of Endogenous Estrogens, Their Precursors, and Metabolites in Endometrial Cancer Patients: Association with Risk and Relationship to Clinical Characteristics. J. Clin. Endocrinol. Metab..

[101] Küçük O., Churley M., Goodman M.T., Franke A., Custer L., Wilkens L.R., St Pyrek J. (1994). Increased Plasma Level of Cholesterol-5 Beta, 6 Beta-Epoxide in Endometrial Cancer Patients. Cancer Epidemiol. Prev. Biomark..

[102] Zeleniuch-Jacquotte A., Shore R.E., Afanasyeva Y., Lukanova A., Sieri S., Koenig K.L., Idahl A., Krogh V., Liu M., Ohlson N. (2011). Postmenopausal Circulating Levels of 2-and 16α-Hydroxyestrone and Risk of Endometrial Cancer. Br. J. Cancer.

[103] Potischman N., Hoover R.N., Brinton L.A., Siiteri P., Dorgan J.F., Swanson C.A., Berman M.L., Mortel R., Twiggs L.B., Barrett R.J. (1996). Case—Control Study of Endogenous Steroid Hormones and Endometrial Cancer. JNCI J. Natl. Cancer Inst..

[104] Colombo I., Lheureux S., Oza A.M. (2020). Summary of Management Guidelines for Endometrial Cancer. Management of Endometrial Cancer.

[105] Hofman Z.L.M. (2020). Bradykinin Driven Inflammation. Ph.D. Thesis.

[106] Fiorito V., Chiabrando D., Petrillo S., Bertino F., Tolosano E. (2019). The Multifaceted Role of Heme in Cancer. Front. Oncol..

[107] Saddoughi S.A., Song P., Ogretmen B. (2008). Roles of Bioactive Sphingolipids in Cancer Biology and Therapeutics. Lipids in Health and Disease.

[108] Cheng M., Bhujwalla Z.M., Glunde K. (2016). Targeting Phospholipid Metabolism in Cancer. Front. Oncol..

[109] Lakhani N.J., Sarkar M.A., Venitz J., Figg W.D. (2003). 2-Methoxyestradiol, a Promising Anticancer Agent. Pharmacother. J. Hum. Pharmacol. Drug Ther..

[110] Li L., Heldin N.-E., Grawé J., Ulmsten U., Fu X. (2004). Induction of Apoptosis or Necrosis in Human Endometrial Carcinoma Cells by 2-Methoxyestradiol. Anticancer Res..

[111] Strand E., Tangen I.L., Fasmer K.E., Jacob H., Halle M.K., Hoivik E.A., Delvoux B., Trovik J., Haldorsen I.S., Romano A. (2019). Blood Metabolites Associate with Prognosis in Endometrial Cancer. Metabolites.

[112] Martínez Y., Li X., Liu G., Bin P., Yan W., Más D., Valdivié M., Hu C.-A.A., Ren W., Yin Y. (2017). The Role of Methionine on Metabolism, Oxidative Stress, and Diseases. Amino Acids.

[113] Knapp P., Baranowski M., Knapp M., Zabielski P., Błachnio-Zabielska A.U., Górski J. (2010). Altered Sphingolipid Metabolism in Human Endometrial Cancer. Prostaglandins Other Lipid Mediat..

[114] Jové M., Gatius S., Yeramian A., Portero-Otin M., Eritja N., Santacana M., Colas E., Ruiz M., Pamplona R., Matias-Guiu X. (2016). Metabotyping Human Endometrioid Endometrial Adenocarcinoma Reveals an Implication of Endocannabinoid Metabolism. Oncotarget.

[115] Eritja N., Jové M., Fasmer K.E., Gatius S., Portero-Otin M., Trovik J., Krakstad C., Sol J., Pamplona R., Haldorsen I.S. (2017). Tumour-Microenvironmental Blood Flow Determines a Metabolomic Signature Identifying Lysophospholipids and Resolvin D as Biomarkers in Endometrial Cancer Patients. Oncotarget.

[116] Rolin J., Maghazachi A.A. (2011). Effects of Lysophospholipids on Tumor Microenvironment. Cancer Microenviron..

[117] Connor K.M., SanGiovanni J.P., Lofqvist C., Aderman C.M., Chen J., Higuchi A., Hong S., Pravda E.A., Majchrzak S., Carper D. (2007). Increased Dietary Intake of ω-3-Polyunsaturated Fatty Acids Reduces Pathological Retinal Angiogenesis. Nat. Med..

[118] Shafiee M.N., Ortori C.A., Barrett D.A., Mongan N.P., Abu J., Atiomo W. (2020). Lipidomic Biomarkers in Polycystic Ovary Syndrome and Endometrial Cancer. Int. J. Mol. Sci..

[119] Njoku K., Chaiserini D., Jones E., Barr C., O’Flynn H., Whetton A., Crosbie E. (2020). Urinary Biomarkers and Their Potential for the Non-Invasive detection of Endometrial Cancer. Front. Oncol..

[120] Shao X., Wang K., Liu X., Gu C., Zhang P., Xie J., Liu W., Sun L., Chen T., Li Y. (2016). Screening and Verifying Endometrial Carcinoma Diagnostic Biomarkers Based on a Urine Metabolomic Profiling Study Using UPLC-Q-TOF/MS. Clin. Chim. Acta.

[121] Zhao H., Jiang Y., Liu Y., Yun C., Li L. (2015). Endogenous Estrogen Metabolites as Biomarkers for Endometrial Cancer via a Novel Method of Liquid Chromatography-Mass Spectrometry with Hollow Fiber Liquid-Phase Microextraction. Horm. Metab. Res..

[122] Roux A., Thévenot E.A., Seguin F., Olivier M.-F., Junot C. (2015). Impact of Collection Conditions on the Metabolite Content of Human Urine Samples as Analyzed by Liquid Chromatography Coupled to Mass Spectrometry and Nuclear Magnetic Resonance Spectroscopy. Metabolomics.

[123] Zegels G., Van Raemdonck G.A.A., Tjalma W.A.A., Van Ostade X.W.M. (2010). Use of Cervicovaginal Fluid for the Identification of Biomarkers for Pathologies of the Female Genital Tract. Proteome Sci..

[124] Njoku K., Crosbie E.J. (2020). Does the Vaginal Microbiome Drive Cervical Carcinogenesis?. BJOG An Int. J. Obstet. Gynaecol..

[125] Ytre-Hauge S., Husby J.A., Magnussen I.J., Werner H.M.J., Salvesen Ø.O., Bjørge L., Trovik J., Stefansson I.M., Salvesen H.B., Haldorsen I.S. (2015). Preoperative Tumor Size at MRI Predicts Deep Myometrial Invasion, Lymph Node Metastases, and Patient Outcome in Endometrial Carcinomas. Int. J. Gynecol. Cancer.

